# Taxonomic history, morphology, evolution, gene pool and stress tolerances of azuki bean and its related wild *Vigna* genetic resources

**DOI:** 10.1270/jsbbs.24008

**Published:** 2024-08-30

**Authors:** Yu Takahashi, Norihiko Tomooka

**Affiliations:** 1 Research Center of Genetic Resources, National Agriculture and Food Research Organization, 2-1-2 Kannondai, Tsukuba, Ibaraki 305-8602, Japan

**Keywords:** azuki bean, genetic resources, taxonomy, *Vigna angularis*

## Abstract

This review compiles information on the morphology, historical taxonomic treatments, species origin, gene pool concept, geographical and ecological habitats, and stress tolerance of the azuki bean (*Vigna angularis*) and related species. Willdenow (1802) first described the azuki bean in the genus *Dolichos*, and Ohwi and Ohashi (1969) finally transferred it to the genus *Vigna*. The genus *Vigna* is currently divided into five subgenera: *Ceratotropis*, *Haydonia*, *Lasiospron*, *Plectrotropis*, and *Vigna*. The subgenus *Ceratotropis* includes the moth bean in section *Aconitifoliae*; the mungbean and black gram in section *Ceratotropis*; and the azuki bean, rice bean, and creole bean in section *Angulares*. The wild species in section *Angulares* generally possess larger standard petal with more prominent appendage, keel petal with longer pocket, pistil with longer style beak compared with those of species in sections *Aconitifoliae* and *Ceratotropis*, and characterized by glabrous mature pod, smooth seed coat, hypogeal germination seed, and cordate primary leaves with petiole. Section *Angulares* currently consists of 13 species. The taxa that cross readily with the azuki beans included in the primary genepool are the wild azuki bean *V. angularis* var. *nipponensis*, *V. nepalensis*, *V. tenuicaulis*, *V. hirtella*, *V. minima*, *V. nakashimae*, and *V. riukiuensis*. These wild species are expected to be used as breeding material against biotic and abiotic stresses.

## Introduction

The genus *Vigna* Savi is an economically important genus from which nine crop species, i.e., *Vigna unguiculata* (L.) Walp. (cowpea), *Vigna subterranea* (L.) Verdc. (Bambara groundnut), *Vigna vexillata* (L.) A. Rich. (tuber cowpea), *Vigna aconitifolia* (Jacq.) Maréchal (moth bean), *Vigna radiata* (L.) Wilczek (mungbean), *Vigna mungo* (L.) Hepper (black gram), *Vigna reflexo-pilosa* Hayata (creole bean), *Vigna umbellata* (Thunb.) Ohwi et H. Ohashi (rice bean), and *Vigna angularis* (Willd.) Ohwi et H. Ohashi (azuki bean), have been domesticated and are cultivated from the tropics to temperate zones for food and feed (Takahashi and Tomooka 2020). This genus includes more than 80 wild species, and some of which have developed high levels of stress tolerances in the course of adaptation to adverse environments, such as drylands (e.g. *Vigna trilobata* (L.) Verdc.), coasts (*Vigna marina* (Burm.) Merr.), swamps (*Vigna luteola* (Jacq.) Benth.), and limestone karst (*Vigna exilis* Tateishi et Maxted) (Tomooka *et al.* 2014). Most wild species of this genus have the same number of chromosomes as crop species (2n = 22) and can be crossed within or between species; therefore, they can be used as genetic resources to impart stress tolerance to *Vigna* crops.

Azuki bean is widely cultivated in Asia and has been introduced to other continents as a cash crop in recent decades (Wang *et al.* 2019). The importance of azuki bean in local industries has led to the development of cultivars with higher yields or biotic and abiotic stress tolerances (Wang *et al.* 2019). In Japan, the planting area of 24,800 ha and the yield of 30,900 tons for azuki bean are the second largest in the legume crops after soybean (*Glycine max* (L.) Merr.) and higher than those of common bean (*Phaseolus vulgaris* L.) and groundnut (*Arachis hypogaea* L.) (Ministry of Agriculture, Forestry and Fisheries 2024). To develop a better understanding of azuki bean and its potential genetic resources, we have compiled information by adding recent publications on their morphology, historical taxonomic treatments, species origin, gene pool concept, geographical and ecological habitats, and stress tolerances.

## Morphological and growth characteristics of azuki bean

**Fig. 1.**
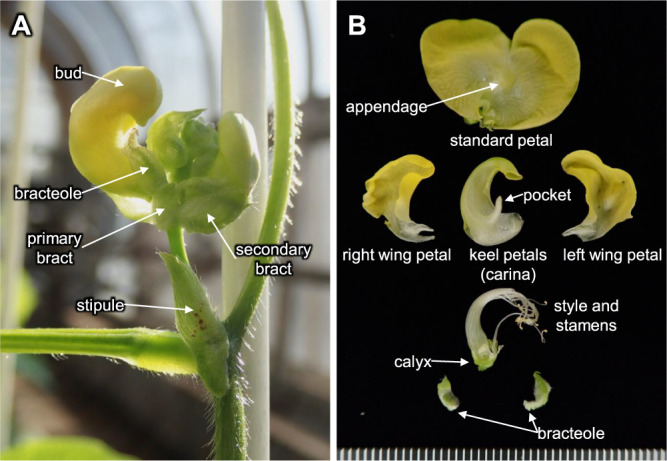
Morphology of azuki bean inflorescence (A) and flower parts (B) of cultivar ‘Syumari’ cultivated in a greenhouse.

The azuki bean shows hypogeal germination in which seeds (cotyledons) remains underground after germination (Tomooka *et al.* 2002b). The plant develops one true leaf each week until the flowering stage. Lateral branches emerge from the first true leaf node after the development of the third true leaf (Chiba 1991). The plant grows in an erect or twining manner to a height of 25–150 cm and has 12–26 main stem nodes (Chiba 1991, Narikawa 2001). The first two primary leaves are simple leaves, then the true leaves consist of three leaflets, and each leaflet is generally 6–10 cm long and 5–8 cm wide with continuous morphological variation, ranging from orbicular with an aspect ratio of 1:1 to linear with an aspect ratio of 15:1, depending on the cultivar or leaf position (Chiba 1991, Ohwi 1953a). The stipule is of a lanceolate shape with a peltate base (Fig. 1A). The stem is green or purple (Chiba 1991). Hair is present on aboveground organs, such as the leaflet and stem, with the amount varying depending on the cultivar (Chiba 1991). Most of the indigenous varieties in China, South Korea, and Japan have hairless pods, but there are some local varieties with hairy pods in Bhutan, Nepal, and Vietnam.

The azuki bean is a quantitative short-day plant with a critical photoperiod of 12–13 h (Kim *et al.* 2014), and the number of days to flowering varies greatly depending on the cultivar and/or environment. Approximately half of the flowers develop into pods, and earlier flowers are more likely to develop into pods (Chiba 1991). The flower bud develops opposite the node of a peduncle (Fig. 1A). The flower bud is covered in one secondary bract and two bracteoles (Fig. 1A, 1B). The flower is yellow, but the shade of yellow varies depending on the cultivar (Chiba 1991) and is about 15–18 mm in diameter consisting of one standard petal, two wing petals, and two keel petals that fuse at the base to form a boat-like structure known as a carina (Fig. 1B) (Ohwi 1953a). The reproductive organ consists of nine adherent stamens, one independent stamen, and one pistil (Fig. 1B) that has hairs on one side; the stigma is lateral, and the style is beaked beyond the stigma (Tomooka *et al.* 2002b).

**Fig. 2.**
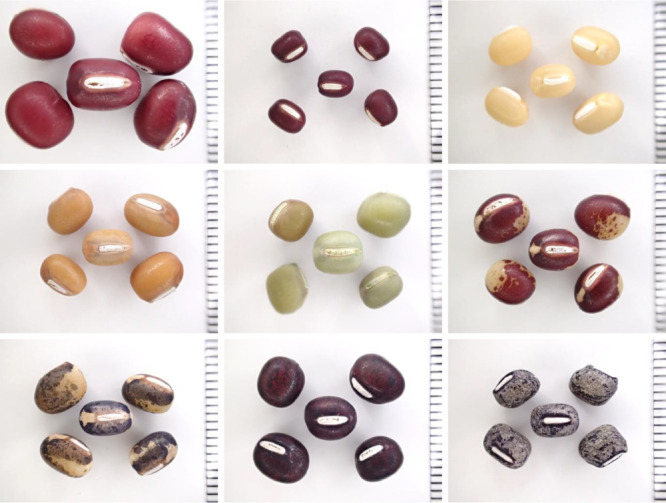
Diversity of size and color in azuki bean seed. The scale on the ruler is in millimeters (mm).

The pod becomes thicker and matures approximately one month after flowering when longitudinal growth reaches a maximum length of 6–10 cm (Chiba 1991, Ohwi 1953a). Seed enlargement lags pod development (Chiba 1991). Similar to the stem, the young pod is green or purple-brown (Chiba 1991). The mature pod varies from dark brown to grayish white depending on the cultivar (Chiba 1991). The small-seed varieties have approximately seven seeds per pod with less curvature and constriction between seeds, while the large-seed varieties have approximately four seeds per pod with greater curvature and constriction between seeds (Chiba 1991). The seed is oval, rounded at both ends, and with a flat hilum (Fig. 2). Although the seed coat is mainly purplish red, the specific shade varies depending on the cultivar among light yellow, light brown, and green; bi-colored and mottled patterns are also found (Fig. 2).

## Historical taxonomic treatments of azuki bean and related species at genus and subgenus rank

The taxonomic treatments of azuki bean and related species have been repeatedly revised. Willdenow (1802) first described the azuki bean in the genus *Dolichos* L. (*Dolichos angularis* Willd.), following which Wight (1909) transferred it to the genus *Phaseolus* L. (*Phaseolus angularis* (Willd.) W. Wight), Ohwi (1953a, 1953b) proposed to transfer it to the genus *Azukia* Takah. ex Ohwi. (*Azukia angularis* (Willd.) Ohwi), and Ohwi and Ohashi (1969) finally transferred it to the genus *Vigna* (*Vigna angularis* (Willd.) Ohwi et H. Ohashi). Verdcourt (1969) proposed subgenus *Ceratotropis* (Piper) Verdc. in genus *Vigna*. Verdcourt (1970) included mungbean, azuki bean and related wild Asian species in the subgenus *Ceratotropis*. Tomooka *et al.* (2002a) subdivided the subgenus *Ceratotropis* into three sections and included the azuki bean and closely related wild species in the section *Angulares* N. Tomooka et Maxted. The azuki bean has some synonyms because it is sometimes confused with the mungbean (*V. radiata*) and black gram (*V. mungo*) (Sacks 1977). To provide a clearer picture of the historical nomenclature of the azuki bean, we have listed below the transition of scientific names and taxonomic treatments of the azuki bean and its close relatives. In addition, Table 1 summarizes the current taxonomic system of the genus *Vigna*, which is divided into five subgenera: *Ceratotropis* (Piper) Verdc., *Haydonia* (Wilczek) Verdc., *Lasiospron* (Benth. emend. Piper) Maréchal, Mascherpa et Stainier, *Plectrotropis* (Schumach.) Bak., and *Vigna* Savi, three of which contain crops. The subgenus *Vigna*, including the cowpea (*V. unguiculata*) and Bambara groundnut (*V. subterranea*) and the subgenus *Plectrotropis*, including the tuber cowpea (*V. vexillata*), are found predominantly in Africa. The subgenus *Ceratotropis* is mainly found in Asia and includes the moth bean (*V. aconitifolia*) in section *Aconitifoliae* N. Tomooka et Maxted; the mungbean and black gram in section *Ceratotropis*; and the azuki bean, rice bean (*V. umbellata*), and creole bean (*V. reflexo-pilosa*) in section *Angulares*.

The historical transition of scientific names and taxonomic treatment of the azuki bean and closely related species are as follows:

1. Linnaeus (1753) described the domesticated common bean as *Phaseolus vulgaris* L., the domesticated mungbean as *Phaseolus radiatus* L., and the domesticated cowpea as *Dolichos unguiculatus* L. in “Species Plantarum.”

2. Jacquin (1770) described the wild hairy cowpea as *Dolichos luteolus* Jacq. in the same genus as Linnaeus’ cowpea.

3. Willdenow (1802) described Engelbert Kämpfer’s “Atsuki (= azuki bean)” as *Dolichos angularis* Willd. and listed 53 species in the genus *Dolichos*.

4. Savi (1824) described the genus *Vigna* Savi using the hairy cowpea as a type species.

5. De Candolle (1825) listed the common bean and mungbean in the genus *Phaseolus* and the cowpea and azuki bean in the genus *Dolichos*.

6. Walpers (1842) transferred *Dolichos unguiculatus* L. to the genus *Vigna* as *Vigna unguiculata* (L.) Walp., which is the currently accepted scientific name of the cowpea.

7. Wight (1909) transferred *Dolichos angularis* Willd. (azuki bean) to the genus *Phaseolus* and described *Phaseolus angularis* (Willd.) W. Wight.

8. Piper and Morse (1914) noted that the azuki bean, mungbean, black gram, and moth bean should be classified in the subgenus *Ceratotropis* in the genus *Phaseolus*.

9. Piper (1926) published a classification system for the American *Phaseolineae* Benth. and classified the annual plant group of the Orient with yellow flowers in section *Ceratotropis* Piper in the genus *Phaseolus*.

10. Ohwi (1937) described the Japanese wild legume “Yabutsuru-azuki (wild ancestor of azuki bean)” as *Phaseolus nipponensis* Ohwi.

11. Ohwi (1953a, 1953b) transferred the azuki bean and “Yabutsuru-azuki” to the new genus *Azukia* Takah. ex Ohwi. as *Azukia angularis* (Willd.) Ohwi and *Azukia angularis* (Willd.) Ohwi var. *nipponensis* (Ohwi) Ohwi.

12. Wilczek (1954) transferred the mungbean from the genus *Phaseolus* to the genus *Vigna* as *Vigna radiata* (L.) Wilczek and listed 39 species in this genus based primarily on the morphology of the stipule and style beak.

13. Hutchinson (1964) considered the genus *Azukia* as a synonym of the genus *Vigna*.

14. Ohwi and Ohashi (1969) recognized the genus *Azukia* as a synonym of the genus *Vigna* and described the azuki bean as *Vigna angularis* (Willd.) Ohwi et H. Ohashi and the wild azuki bean as *Vigna angularis* (Willd.) Ohwi et H. Ohashi var. *nipponensis* (Ohwi) Ohwi et H. Ohashi.

15. Verdcourt (1969) transferred section *Ceratotropis* from the genus *Phaseolus* to the genus *Vigna* and described it as the subgenus *Ceratotropis* (Piper) Verdc.

16. Verdcourt (1970) proposed a narrower definition for the genus *Phaseolus* and a broader definition for the genus *Vigna* consisting of eight subgenera. He classified the cowpea in the subgenus *Vigna* and the azuki and mungbean in the subgenus *Ceratotoropis* (Piper) Verdc.

17. Maréchal *et al.* (1978) subdivided the genus *Vigna* into seven subgenera and listed 17 species in the subgenus *Ceratotropis*, including the azuki bean.

18. Tomooka *et al.* (2002b) described 21 species in the subgenus *Ceratotropis* and subdivided them into three sections: *Aconitifoliae* N. Tomooka et Maxted, *Ceratotropis*, and *Angulares* N. Tomooka et Maxted, and classified the azuki bean in section *Angulares*.

19. Thulin *et al.* (2004) separated the species in the subgenus *Macrorhynchus* Verdc. found in Africa from the genus *Vigna*, classifying them as the newly described genus *Wajira* Thulin.

20. Delgado-Salinas *et al.* (2011) transferred species in the subgenus *Sigmoidotropis* (Piper) Verdc. from the Americas to other genera.

21. Pasquet and Padulosi (2013) proposed transferring species in sections *Macrodontae* Harms, *Reticulatae* Verdc., and *Catiang* (DC.) Verdc in the subgenus *Vigna*, including the cowpea, to the subgenus *Plectrotropis*.

## Key characters of genera *Vigna* and *Phaseolus*

As the key characters that distinguish species in the genera *Vigna* and *Phaseolus*, Bentham (1865) focused on the curvature of keel petal, Wilczek (1954) on the leaflet and style beak, Hepper (1958) on the pod septal wall, Tourneur (1958) on the petiole of the primary leaf, and Verdcourt (1970) on the pollen grain morphology. Following this, Maréchal *et al.* (1978) graphically demonstrated the variation in the stipule, tubercle on the inflorescence branch, keel petal, pistil, and style beak between these two genera (see Fig. 2.5 in Tomooka *et al.* 2002b). Thereafter, while the number of subgenera in the genus *Vigna* decreased, the number of *Phaseolus* species increased from 17 in Maréchal *et al.* (1978) to 76 in Freytag and Debouck (2002). Although some of the above characters are effective at distinguishing the genera *Vigna* from *Phaseolus* in the current classification system, there is a need to reconsider the key characters based on the observation of newly included and excluded species for each genus.

## Evolution of subgenera *Vigna*, *Plectrotropis*, and *Ceratotropis*

**Fig. 3.**
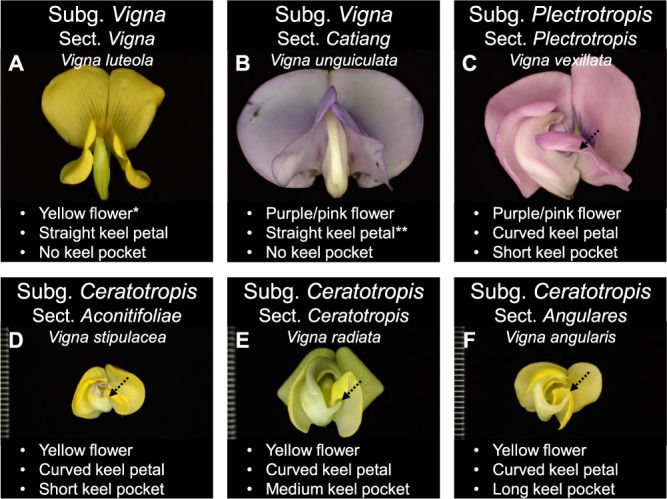
Flower of subgenera *Vigna* (A, B), *Plectrotropis* (C), and *Ceratotropis* (D–F). The arrow indicates the keel pocket. *Vigna parkeri Baker (subg. *Vigna*) includes blue, purple, pink, and yellow flowers as intraspecific variations (Maxted *et al.* 2004). **Vigna membranacea A. Rich. (subg. *Vigna*) includes a subspecies with a curved keel petal and another with a straight keel petal (personal observation by Y.T. 2019).

Tateishi and Ohashi (1990) considered that the subgenus *Plectrotropis* evolved from the subgenus *Vigna*, and then the subgenus *Ceratotropis* evolved from the subgenus *Plectrotropis*. This hypothesis is based on the flower morphology becoming more complex from the subgenus *Vigna* through subgenus *Plectrotropis* to subgenus *Ceratotropis*. The subgenus *Vigna* has a simple symmetrical straight keel without a pocket (Fig. 3), subgenus *Plectrotropis* has a slightly curved keel with a pocket (Fig. 3), and subgenus *Ceratotropis* has a more curved keel with a more prominent pocket (Fig. 3, see Fig. 2.2 in Tomooka *et al.* 2002b).

Kang *et al.* (2014) published a phylogenetic tree based on next generation sequencing (NGS) data. This phylogenetic tree supported Tateishi and Ohashi’s hypothesis described above, with the Bambara groundnut being placed in the subgenus *Vigna* (section *Vigna*) at the base and subgenera *Plectrotropis* and *Ceratotropis* subsequently diverging. However, this phylogenetic tree did not include cowpea, an important crop in the subgenus *Vigna* (section *Catiang*). Phylogenetic trees based on partial sequencing of chloroplast DNA containing the cowpea have shown that the position of the cowpea contradicted Tateishi and Ohashi’s hypothesis (Delgado-Salinas *et al.* 2011, Takahashi *et al.* 2016). In these two phylogenetic trees, the cowpea (section *Catiang* in subgenus *Vigna*) was more closely related to the tuber cowpea (*V. vexillata*, subgenus *Plectrotropis*) rather than to the hairy cowpea (*V. luteola*, section *Vigna* in subgenus *Vigna*). For this reason, Takahashi *et al.* (2016) suggested that it would be appropriate to raise the rank of section *Catiang*, including the cowpea, to a new subgenus within the genus *Vigna*. However, these authors did not carry out the taxonomic revision themselves because only few species in subgenera *Vigna* and *Plectrotropis* were analyzed. Pasquet and Padulosi (2013) reported that *Vigna* species with pink flowers (sections *Macrodontae*, *Reticulatae*, and *Catiang* in subgenus *Vigna*) should be transferred to the subgenus *Plectrotropis* (Fig. 3). Based on the close molecular phylogenetic positions, it is worth promoting the utilization of the tuber cowpea, which has large intraspecific variation, as a genetic resource for the cowpea.

## Evolution of sections Aconitifoliae, Ceratotropis, and Angulares

All species of the subgenus *Ceratotropis*, which mainly inhabit Asia, possess a yellow flower, keel petal curved 160 to 360° to the left, and a peltate stipule at the base. Tomooka *et al.* (2002b) listed 21 species in the subgenus *Ceratotropis* and proposed a classification system to place them into three sections based on their geographical distribution and morphological characteristics. Since then, six new species from India have been described, with 27 species currently reported (Table 2). However, we have not been able to obtain the seeds of four species (*Vigna yadavii* S. P. Gaikwad, Gore, S. D. Randive et Garad, *Vigna konkanensis* Latha, K. V. Bhat, I. S. Bisht, Scariah, K. J. John et Krishnaraj, *Vigna pandeyana* Gore, S. P. Gaikwad et S. D. Randive, *Vigna sathishiana* Balan et Predeep) from any genebank or other source to investigate the morphology and molecular phylogeny. Therefore, the status of these independent species remains unknown.

Section *Aconitifoliae* species mainly inhabit arid areas of South Asia, possess a creeping stem, pedately parted leaflet, standard petal without appendage, keel petal with short pocket, short style beak, small flower, and a seed that is epigeal when germinated. As an exception, *Vigna khandalensis* (Santapau) Sundararagh. et Wadhwa, which is found in the tropical rainforest climate zone of the Western Ghats, India, grows in an erect manner like a crop plant, despite being a wild species. The moth bean (*V. aconitifoliae*), which is the most heat- and drought-tolerant crop in the subgenus *Ceratotropis*, is included in this section.

*Ceratotropis* species mainly inhabit South and Southeast Asia. The mungbean (*V. radiata*) and black gram (*V. mungo*) are included in this section. The wild species in this section possess a twining stem, flower larger than that of section *Aconitifoliae*, standard petal with an appendage, keel petal with longer pocket, longer style beak, setose mature pod, rough seed coat (dull), seed that is epigeal when germinated, and sessile primary leaf. In section *Ceratotropis*, four new species have been discovered since Tomooka *et al.* (2002b), with seven species currently recognized (Table 2). Among these new species, Takahashi *et al.* (2016) analyzed the morphology and phylogeny of *Vigna sahyadriana* Aitawade, K. V. Bhat et S. R. Yadav. This species showed intermediate morphology between wild mungbean and wild black gram but was more closely related to the black gram on the phylogenetic tree. In addition, while the original description of *V. sahyadriana* noted that its habitat was limited to the Northern Western Ghats, India (Aitawade *et al.* 2012), Takahashi *et al.* (2017) found this species on a rocky slope beside a road in a valley at an altitude of 2,008 m in western Nepal (JP257532), indicating that the geographical distribution of this species is broader than originally thought.

Section *Angulares* species inhabit humid environments from Southeast to East Asia. The azuki bean (*V. angularis*), rice bean (*V. umbellata*), and creole bean (*V. reflexo-pilosa*) are included in this section. The wild species in this section possess a twining stem, flower generally larger than that of sections *Aconitifoliae* and *Ceratotropis*, standard petal with appendage, keel petal with longer pocket, longer style beak, glabrous mature pod, smooth seed coat, hypogeal germination seed, and cordate primary leaves with the petiole. As an exception, *Vigna trinervia* (B. Heyne ex Wight et Arn.) Tateishi et Maxted, which is found from Tanzania to Madagascar in the west and from southern India to Indonesia in the east, possesses the morphological characteristics of section *Ceratotropis*, such as setose mature pod and rough seed coat (dull). Tomooka *et al.* (2002b) classified this species into section *Angulares* based on its hypogeal germination and cordate primary leaf with petiole and considered this species to have intermediate morphology between that of sections *Ceratotropis* and *Angulares*.

In recent years, phylogenetic trees based on NGS data of nuclear genomes (Kang *et al.* 2014) and partial DNA sequences of chloroplast genomes (Delgado-Salinas *et al.* 2011, Javadi *et al.* 2011, Takahashi *et al.* 2016) have been reconstructed to clarify the phylogenetic relationships of the genus *Vigna*. As a result, section *Angulares*, including the azuki bean, was clustered into a monophyletic group, but species of sections *Aconitifoliae* and *Ceratotropis* were clustered into one monophyletic group rather than two groups. Thus, the subgenus *Ceratotropis* is composed of two monophyletic groups. At the same time, *Vigna trinervia* was clustered into a group combining sections *Aconitifoliae* and *Ceratotropis* but not into section *Angulares*.

The current taxonomy does not always recognize monophyletic groups solely as taxa. Therefore, we divided the subgenus *Ceratotropis* into three sections because this classification system based on geographical distribution, ecology, and morphology provides important information when identifying species in the field or considering the utilization of wild species as genetic resources. However, based on the DNA data, it is considered that *V. trinervia* should be transferred from section *Angulares* to section *Ceratotropis* as a species with some characteristics of section *Angulares*.

## Species in section *Angulares*

**Fig. 4.**
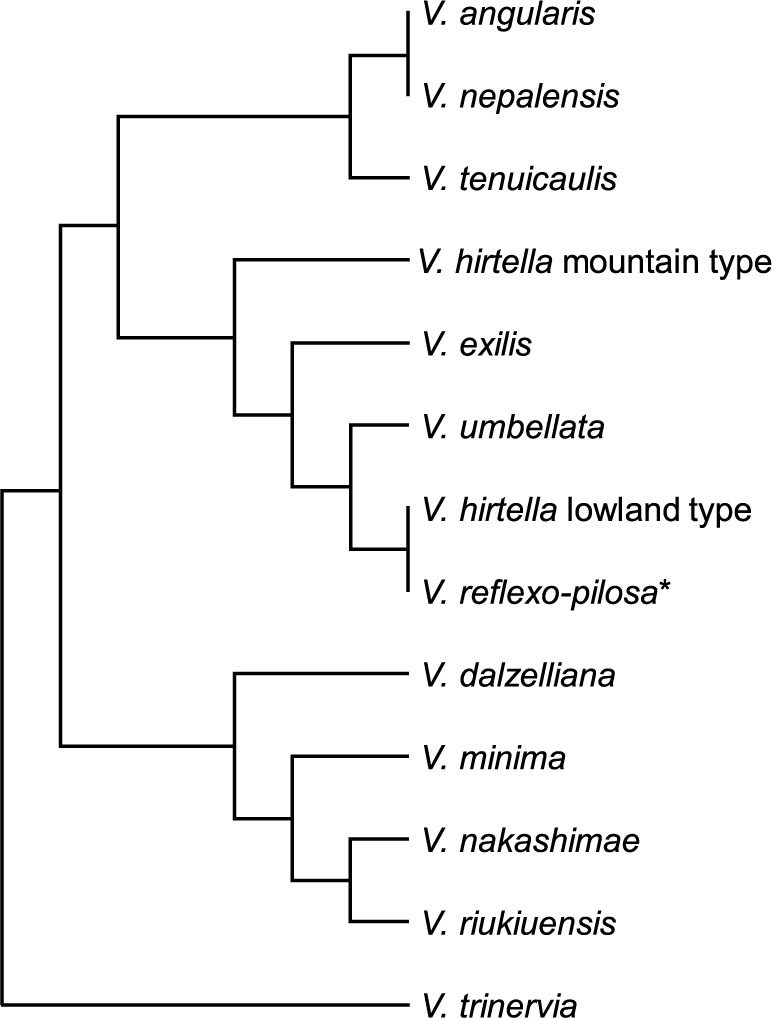
Phylogenetic tree based on the rDNA-ITS of section *Angulares* in subgenus *Ceratotropis* in genus *Vigna*. The data include unpublished data along with data from Takahashi *et al.* (2016). This phylogenetic tree was shown only in topology and compressed per clade for each species. *Tetraploid species.

Section *Angulares*, including the azuki bean, currently consists of 13 species (Table 2). We present a modified phylogenetic tree based on the rDNA-ITS of Takahashi *et al.* (2016) for 12 species, excluding *Vigna yadavii* S. P. Gaikwad, Gore, S. D. Randive et Garad (Fig. 4).

The wild azuki bean *Vigna angularis* var. *nipponensis* has been found in Japan, Korean Peninsula, China, Laos, Myanmar, Bhutan, Nepal, and India (Takahashi *et al.* 2019, Tomooka *et al.* 2002b, 2006b, 2020). Tateishi and Maxted (2002) described *V. nepalensis* Tateishi et Maxted as a new species based on field surveys in eastern Nepal. They noted 10 key characters to distinguish *V. nepalensis* from the wild azuki bean, including differences in the length of the hilum, shape of the bracteole, and pubescence of the inflorescence rachis as the most useful. They noted that *V. nepalensis* is also found in Bhutan and India (Sikkim, Darjeeling, and Assam), and both species segregate according to altitude in eastern Nepal, with the wild azuki bean inhabiting higher altitude areas and *V. nepalensis* inhabiting lower altitude areas. However, phylogenetic differentiation was not observed between *V. nepalensis* and *V. angularis* var. *nipponensis* in the rDNA-ITS tree (Fig. 4). In addition, sympatric plants with wild azuki bean and *V. nepalensis* key characters were found in the mountains of northern Myanmar (identified as *V. angularis* var. *nipponensis*, Tomooka *et al.* 2020). Nevertheless, as large-scale chloroplast DNA analyses have found nucleotide polymorphisms between individuals of both species (Lee *et al.* 2023), population genomics may reveal differences between *V. angularis* and *V. nepalensis*.

*Vigna tenuicaulis* N. Tomooka et Maxted is the species most closely related to the azuki bean besides *V. nepalensis* (Fig. 4). This species was described by Tomooka *et al.* (2002a) as a new species based on a holotype collected in northern Thailand, with later surveys conducted in Myanmar and Laos finding more habitats (Tomooka *et al.* 2006b, 2020). Since this species inhabits lower altitudes in Myanmar and Laos than the wild azuki bean, it is a promising genetic resource to improve the heat tolerance of the azuki bean.

*Vigna hirtella* Ridl. was described based on a specimen (lectotype: K000900684) collected in the lowlands of Kelantan, Malay Peninsula (Ridley 1920). Although Tomooka *et al.* (2002a, 2002b) noted key characters to distinguish *V. hirtella* from other species, the National Agriculture and Food Research Organization (NARO) Genebank accessions, which were classified as *V. hirtella* based on the key characters, were separated to two clades in some molecular phylogenetic trees (Chankaew *et al.* 2014, Seehalak *et al.* 2006, Tomooka *et al.* 2002a, 2002b, 2006a). This indicates that differentiated plants exist that should be treated as independent species even though they are morphologically similar to *V. hirtella*. In Fig. 4, two groups on the phylogenetic tree are shown as *V. hirtella* mountain type and *V. hirtella* lowland type. The *V. hirtella* mountain type includes eight accessions collected at relatively high altitudes in Thailand (JP108515, JP108562), Laos (JP220131, JP220135, JP224436, JP226635, JP226669), and Nepal (JP257571); the *V. hirtella* lowland type includes six accessions collected at relatively low altitudes on the Malay Peninsula (JP 108851) and in Thailand (JP108566, JP 226687, JP205885), Laos (JP226687), and Sri Lanka (JP218935). Evaluation of useful traits and revision of taxonomic treatment in these groups are needed in future studies. In any case, there is no doubt that future research will uncover new species.

*Vigna exilis* has been described as having adapted to the limestone mountains of Thailand (Tateishi and Maxted 2002). This species has morphological characteristics that are not found in other wild species in section *Angulares*, such as an extremely elongated seed and a very thin light brown pod. Accession JP210644 (CED99T-9) was found at the lower site of the limestone hill and was initially identified as *V. umbellata* (Tomooka *et al.* 2000). The stem and leaf of the plant have nearly no hairs, which is characteristic of *V. exilis*. However, an author (N.T.) identified this plant as *V. umbellata* based on the seed morphology which is very similar to *V. umbellata*, with a much larger seed size (1.7 g/100 seeds) than other *V. exilis* accessions (0.4–0.5 g/100 seeds). Later, Takahashi *et al.* (2015a) demonstrated experimentally that JP210644 is a hybrid-descendant between *V. exilis* and wild *V. umbellata*. The hybrids can continue to inhabit limestone mountains since they acquired earlier flowering and higher drought tolerance from *V. exilis* compared with the nearby wild *V. umbellata* accessions.

*Vigna umbellata* includes the rice bean, an important crop in mountainous slash-and-burn farming fields in continental Southeast Asia, and its wild ancestor inhabits continental Southeast Asia. Escaped natural populations have been found in the Americas (https://www.discoverlife.org/mp/20m?kind=Vigna+umbellata). We believe that its domestication occurred in continental Southeast Asia (Tian *et al.* 2013).

*Vigna reflexo-pilosa*, which is the only tetraploid species (2n = 44) in the genus *Vigna*, includes domesticated taxa (*V. refloxo-pilosa* var. *glabra*, syn. *Vigna glabrescens* Maréchal, Mascherpa et Stainier) from Vietnam (JP105818, JP105819), the Philippines (JP109684), and Angola (JP 42084). Wild plants are distributed from Southeast Asia to the Pacific Islands, and to the Ryukyu Islands in Japan. *V. trinervia* and *V. hirtella* hill types are thought to be genome donors to *V. reflexo-pilosa* based on their phylogenetic relationships (Chankaew *et al.* 2014, Kang *et al.* 2014, Ye and Yamaguchi 2007).

*Vigna dalzelliana* (Kuntze) Verdc. has been found only in southern India and Sri Lanka based on a herbarium survey of the collections at the Royal Botanic Gardens, Kew and the British Museum in London, the Muséum national d'histoire naturelle in Paris, Meise Botanic Garden in Belgium, and Rijksherbarium in the Netherlands (Tomooka *et al.* 2002b). However, Tomooka *et al.* (2003) reported that they had collected *V. dalzelliana* plants from southern Myanmar and mentioned that it is necessary to clarify the phylogenetic relationship between *V. dalzelliana* plants in Sri Lanka and those in southern Myanmar. Subsequently, John *et al.* (2009) reported *V. dalzelliana* on the Andaman Islands between Sri Lanka and southern Myanmar. Takahashi *et al.* (2016) revealed through phylogenetic analysis, comparing with the Indian accession of *V. dalzelliana* (JP235419), that the plants collected from southern Myanmar (JP210811) by Tomooka *et al.* (2003) were *V. dalzelliana*, showing its geographical distribution extended to southern Myanmar.

*Vigna minima* (Roxb.) Ohwi et H. Ohashi, *V. nakashimae* (Ohwi) Ohwi et H. Ohashi, and *V. riukiuensis* (Ohwi) Ohwi et H. Ohashi are all closely related and are referred to as the *V. minima* complex (Yoon *et al.* 2000). We considered that *Vigna minima*, which had inhabited continental Southeast Asia, was differentiated into *V. nakashimae* through isolation in Korean Peninsula and western Kyushu, Japan, and into *V. riukiuensis* through adaptation to the coast from Taiwan to Okinawa Prefecture, Japan. *Vigna minima* is widely found in Southeast Asia (Tomooka *et al.* 2002b), especially in Cambodia as its center of diversity (Takahashi *et al.* 2014, 2015b, Tomooka *et al.* 2012, 2013). On the Iki and Goto Islands in Nagasaki Prefecture, Japan, *Vigna nakashimae* inhabits coastal quays and possesses high salt tolerance along with *V. riukiuensis*, as described in section Abiotic stresses of this review.

*Vigna trinervia* should be classified in section *Ceratotropis* based on the DNA data, as mentioned above. This species has been found in a wide geographical range, including Madagascar, southern India, Sri Lanka, continental Southeast Asia, and East Timor (Tomooka *et al.* 2002b), and is a maternal genome donor to *V. reflexo-pilosa*, the tetraploid species described above (Chankaew *et al.* 2014, Kang *et al.* 2014, Ye and Yamaguchi 2007).

*Vigna yadavii*, which was described as a new species by Gaikwad *et al.* (2014), was classified into section *Angulares* based on its standard petal with appendage, seed with hypogeal germination, and petiolate primary leaf by Takahashi and Tomooka (2020). However, we could not include this species in Fig. 4 because we were unable to obtain the seeds from any genebank or other source to perform phylogenetic analysis. The key characteristic to distinguish this species from *V. dalzelliana* is the presence of a subterranean cleistogamous flower, but the intraspecific variation of this character has never been investigated for *V. dalzelliana*. Takahashi *et al.* (2017) observed that wild *V. angularis* var. *nipponensis* and *V. hirtella* mountain type possess bi-morphic pods and short pods close to or under the ground surface, while the same plants produce longer pods on the aerial shoot in the mountainous areas of Nepal, and considered that this character was acquired in the process of adaptation to the sloped environment (Takahashi *et al.* 2017). Morphological and phylogenetic analyses of *V. yadavii* and *V. dalzelliana* are anticipated to validate the taxonomic rank of *V. yadavii*.

## Origin of wild *Vigna angularis*

**Fig. 5.**
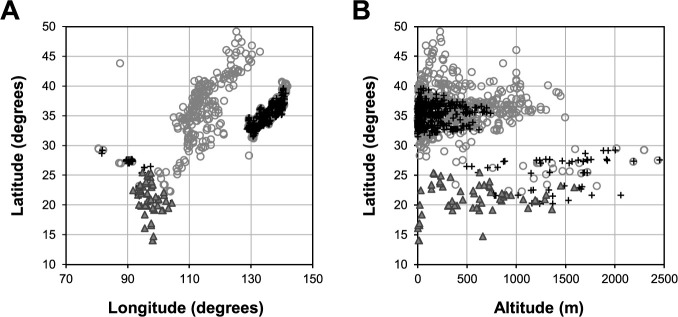
Scatter plot of longitude and latitude (A) and altitude and latitude (B) of the collection sites for domesticated azuki bean *V. angularis* var. *angularis* (○), wild azuki bean *V. angularis* var. *nipponensis* (+), and *V. tenuicaulis* (▲) accessions conserved in the NARO Genebank.

The wild azuki bean *V. angularis* var. *nipponensis* has been found at altitudes lower than 700 m in Japan (latitude between 30° and 40°), whereas it has been found at altitudes higher than 500 m to 2500 m in Laos, Myanmar, Bhutan, and Nepal (latitudes between 20° and 30°) (Fig. 5). The northernmost NARO Genebank (GB) accession is JP225126, which was collected at a latitude of 39.58°, longitude of 140.52°, and altitude of 60 m in Akita Prefecture, Japan. The southernmost GB accession is JP226665, which was collected from a latitude of 20.22°, a longitude of 103.97°, and an altitude of 1,370 m in Xam Nua in Laos. The westernmost GB accession is JP 259888, which was collected from a latitude of 28.64°, longitude of 81.62°, and altitude of 1,650 m in the Himalayas, Nepal. The highest altitude GB accession is JP245937, which was collected from a latitude of 27.54°, longitude of 89.65°, and altitude of 2,455 m in Bhutan. Since most of these areas are classified as temperate zones (C) in the Köppen climate classification, we consider that *V. angularis* var. *nipponensis* has developed cold tolerance and is unable to inhabit tropical environments.

In contrast, *V. tenuicaulis*, which is closely related to *V. angularis* var. *nipponensis*, is found mainly at altitudes of 1,500 m or lower in areas with latitudes of 25° or lower, including Thailand, Myanmar, and Laos (Fig. 5). The southernmost GB accession of this species is JP 210814, which was collected from a latitude of 12.47°, longitude of 98.60°, and altitude of 15 m in Myanmar. This is a lowland area at the base of the Malay Peninsula and is classified as a tropical monsoon climate (Am) in the Köppen climate classification. Therefore, we consider that *V. tenuicaulis* has developed heat tolerance gene(s) which could be utilized for azuki bean improvement, and it is unable to inhabit cool environments.

Based on the present habitats and geographical distribution of the two abovementioned species, we consider that a common ancestor that inhabited northern Myanmar was differentiated into *V. angularis* var. *nipponensis* through adaptation to the cool environments of mountainous areas, and *V. tenuicaulis* adapted to the tropical environments of lowland areas. Mountainous areas of northern Myanmar have cool environments and short warm periods; therefore, cold tolerance and/or early flowering periods are required to inhabit these areas. Thereafter, *V. angularis* var. *nipponensis* could spread into analogous cool areas along the Himalayas (Bhutan, Nepal, and India) and then northeastward to the western and lowland areas of East Asia (China, the Korean Peninsula, Taiwan, and Japan). Similar agro-ecological cultures known as “The Broadleaved Evergreen Forest Culture” are recognized in these areas (Tomooka 2009).

## Gene pool and genetic resources of the azuki bean

The gene pool concept based on the cross-compatibility of wild species with the target crop is useful for plant breeding (Harlan and de Wet 1971). Maxted *et al.* (2006) specifically redefined the gene pool concept with the aim of more efficiently utilizing the wild relatives of crops; GP-1 includes “taxa that cross readily with the crop (or can be predicted to do so based on their taxonomic relationships), yielding (or being expected to yield) fertile hybrids with good chromosome pairing, making gene transfer through hybridization simple.” GP-2 is defined as “taxa that will successfully cross with the crop (or can be predicted to do so based on their taxonomic relationships), but yield (or would be expected to yield) partially or mostly sterile hybrids with poor chromosome pairing, making gene transfer through hybridization difficult.” GP-3 is defined as “taxa that can be crossed with the crop (or can be predicted to do so based on their taxonomic relationships), but hybrids are (or are expected to be) lethal or completely sterile. Special breeding techniques, some yet to be developed, are required for gene transfer.”

**Fig. 6.**
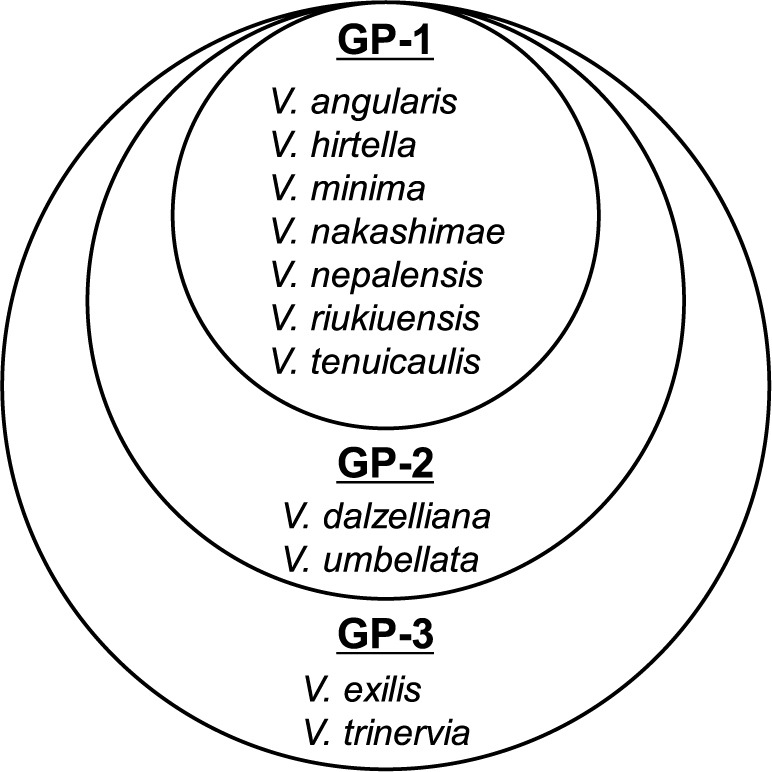
Gene pool concept for the azuki bean. Section *Angulares* taxa are examined and included. GP-1 includes “taxa that cross readily with the crop (or can be predicted to do so based on their taxonomic relationships), yielding (or being expected to yield) fertile hybrids with good chromosome pairing, making gene transfer through hybridization simple.” GP-2 is defined as “taxa that will successfully cross with the crop (or can be predicted to do so based on their taxonomic relationships), but yield (or would be expected to yield) partially or mostly sterile hybrids with poor chromosome pairing, making gene transfer through hybridization difficult.” GP-3 is defined as “taxa that can be crossed with the crop (or can be predicted to do so based on their taxonomic relationships), but hybrids are (or are expected to be) lethal or completely sterile. Special breeding techniques, some yet to be developed, are required for gene transfer” (Maxted *et al.* 2006).

Since Tomooka *et al.* (2002b, 2005, 2011) proposed a gene pool concept for the azuki bean, there have been no reports of new combinations of interspecific hybridization with the azuki bean. Here, we present a modified conceptual diagram of the azuki bean gene pool according to the definition of Maxted *et al.* (2006) (Fig. 6). The modifications from Tomooka *et al.* (2011) are that *V. hirtella*, which was placed between GP-1 and GP-2, is now placed in GP-1 for simplicity and *V. dalzelliana* is newly placed in GP-2. GP-1 consists of *V. angularis*, *V. nepalensis*, *V. tenuicaulis*, *V. hirtella*, *V. minima*, *V. nakashimae*, and *V. riukiuensis*, for which fertile hybrids were obtained by crossing with the azuki bean. GP-2 consists of *V. dalzelliana* and *V. umbellata*. F_1_ hybrids between the azuki bean and *V. dalzelliana* could be obtained, but they were sterile and could not produce F_2_ seeds. BC_1_ seeds were obtained by backcrossing with the azuki bean. No F_1_ hybrid seeds could be obtained by direct crossing between the azuki bean and *V. umbellata*. However, using bridge species such as *V. minima*, *V. riukiuensis*, *V. nakashimae*, *V. nepalensis*, and *V. tenuicaulis*, gene transfer from *V. umbellata* to the azuki bean is expected to be possible (Tomooka *et al.* 2002b). GP-3 consists of *V. trinervia* and *V. exilis* in which F_1_ hybrid seeds with the azuki bean were obtained, but the hybrid plants could not grow.

### Genetic resources of azuki bean and its breeding in Japan

Table 3 shows the genebanks that preserve the domesticated and/or wild azuki bean. The origins (collection countries) of domesticated azuki bean accessions are Nepal, Bhutan, Vietnam, Myanmar, Laos, China, South Korea, and Japan. These accessions include indigenous varieties and breeding lines.

Domesticated azuki bean varieties that are grown in Hokkaido, Japan (main azuki bean producing area, which is cool areas at latitudes of 40° or higher) often require cold tolerance (Chiba 1991). Commercial cultivation is also possible in the Columbia Basin of Washington State in the United States at latitudes of 46–48° (Lumpkin and McClary 1994), and the accessions of the domesticated azuki bean were collected at a latitude 49.17° in Heilongjiang Province, China (Fig. 5). In Nepal, Bhutan, and Vietnam, domesticated azuki bean accessions have been collected in cool plateau areas at latitudes of 20° to 30° and altitudes of 1,000 m or higher, which is similar to the geographic range of the wild azuki bean (Fig. 5). Accession JP108240 was collected at an altitude of 2,800 m in Pangum, Nepal, which is not included in Fig. 5 due to the lack of latitude and longitude information of this accession. Along with the accessions from high-latitude areas, those from low latitudes but at high altitudes are considered promising genetic resources to improve the cold tolerance of the azuki bean.

Here, we provide an overview of the Japanese azuki bean cultivar ‘Syumari’ (or ‘Shumari’), which was developed by imparting multiple disease resistance to the popular Japanese cultivar ‘Erimo-shozu.’ Sakai *et al.* (2015, 2016) conducted whole genome sequencing and constructed a genome database for ‘Syumari.’ ‘Erimo-shozu,’ which is the most widely cultivated modern Japanese azuki bean cultivar, was developed by crossing the female parent ‘Kotobuki-azuki’ and the male parent ‘Toiku No.77’ (Murata *et al.* 1985). ‘Takara-azuki,’ selected from indigenous varieties in 1959, was the most widely cultivated for more than 20 years due to its stable yield, lodging resistance, and high quality. However, ‘Takara-azuki’ was overtaken by ‘Erimo-shozu,’ which became a recommended cultivar in Hokkaido in 1981 (Chiba 1991). ‘Syumari’ was developed by crossing two hybrid lines [(‘Erimo-shozu’ × ‘Urasa’) × (‘Erimo-shozu’ × ‘Kuro-azuki’)] and became a recommended cultivar in Hokkaido in 2000 (Fujita *et al.* 2002). ‘Urasa’ from Shimane prefecture is resistant to Phytophthora stem rot disease (*Phytophthora vignae*), and ‘Kuro-azuki’ from Okayama prefecture was resistant to azuki bean brown stem rot disease (BSR) by *Cadophora gregata* and azuki bean *Fusarium* wilt (AFW) by *Fusarium oxysporum*. However, ‘Syumari’ accounted for only 3% of azuki bean cultivation in Hokkaido in 2017 since stable yields could not be secured due to an unstable pod set caused by low and/or high temperatures (temperature-induced sterility) during the flowering period (Nagaoka *et al.* 2020). For this reason, ‘Erimo 167’ was developed in 2017, which was developed with the disease resistances of ‘Syumari’ to ‘Erimo-shozu’ by seven continuous backcrossing events using the DNA marker “Pga1” linked with resistance to BSR by Suzuki *et al.* (2013) (Sato 2018).

### Genebanks conserving the subgenus *Ceratotropis*

The NARO Genebank conserves the most abundant genetic resources of wild and domesticated species of the subgenus *Ceratotropis*, including the azuki bean. Table 4 shows the number of accessions and the origin (collection country) for the 12 species excluding *V. yadavii* in the NARO Genebank. In Table 4, *Vigna angularis*, including the domesticated azuki bean, has the highest number of accessions at 2,248 followed by *V. umbellata*, including the domesticated rice bean, with 461. These accessions primarily originated in Asia, although *V. umbellata* was also collected from the United States and African countries (Table 4). The NARO Genebank is still conducting research on the collection, preservation, and evaluation of genetic resources in the subgenus *Ceratotropis*.

## Stress tolerances of wild *Vigna* species

Wild species in *Vigna* are expected to be used as breeding materials against biotic and abiotic stresses (Tomooka *et al.* 2011, 2014).

### Biotic stresses

Kondo and Tomooka (2012) evaluated the disease resistance of 252 accessions from 26 *Vigna* species to find new resistant material to race 1, 2, and 3 of azuki bean BSR and race 3 of AFW, which are notorious soil-borne diseases affecting azuki bean cultivation in Hokkaido, Japan. They found that 28 accessions of four species (*V. angularis* var. *nipponensis*, *V. hirtella*, *V. minima*, and *V. tenuicaulis*) were resistant to all races of the above two diseases (disease response group D). These four species are included in GP-1 of the azuki bean (Fig. 6), and thus can be used for azuki bean breeding. In addition, 144 resistant accessions categorized as new disease response groups (groups E, F, G, and H) were found. These accessions are likely to contain novel resistance genes.

Kushida *et al.* (2013) evaluated pest resistance in 342 accessions from eight *Vigna* species that can be crossed with the azuki bean to find material resistant to the soybean cyst nematode (*Heterodera glycines*), a serious pest of soybean (*Glycine max* (L.) Merr.) and azuki bean cultivation in Hokkaido, Japan. This resulted in the identification of four accessions of *V. minima* (JP205886, JP205891, and JP210806) and *V. nakashimae* (JP107879), which are highly resistant to all races occurring in Japan (races 1, 3, and 5). Since previously reported cyst nematode-resistant soybean accessions are susceptible to race 5, it is considered that the resistance mechanism and responsible gene(s) in the four wild *Vigna* accessions are novel.

### Abiotic stresses

As previously mentioned in this review, *V. tenuicaulis* seems to represent promising heat-tolerant breeding materials for azuki bean development since its accession JP210817 inhabited lowland areas at the base of the Malay Peninsula. In future research, the evaluation of cold and heat tolerance using *V. angularis* var. *nipponensis* and *V. tenuicaulis* may lead to the development of azuki bean that are suitable for cultivation in a wide range of areas.

**Fig. 7.**
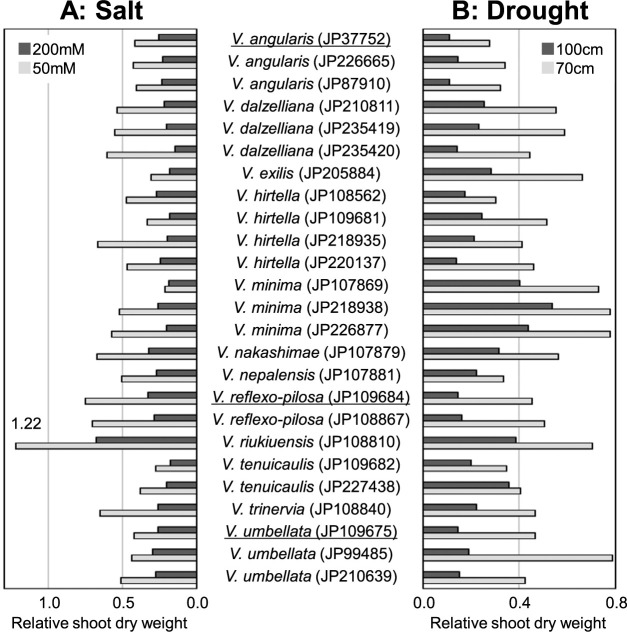
Drought and salt tolerance in section *Angulares*. Only data for section *Angulares* were extracted from Iseki *et al.* (2016) and Iseki *et al.* (2018). Stress tolerance was evaluated by relative dry weight. Cultigens are underlined.

Recently, systematic evaluations of salt and drought tolerance in the genus *Vigna* with an emphasis on the subgenus *Ceratotropis* were carried out (Iseki *et al.* 2016, 2018, Yoshida *et al.* 2016). Fig. 7 shows the levels of salt and drought tolerance in accessions of section *Angulares* species extracted from Iseki *et al.* (2016, 2018). Yoshida *et al.* (2016) evaluated salt tolerance in 219 accessions from seven section *Angulares* species that can be crossed with the azuki bean and revealed high salt tolerance in *V. riukiuensis* and *V. nakashimae*. They also showed that *V. riukiuensis* is a “Na^+^ includer type” isolating sodium ions in the cell, while *V. nakashimae* is a “Na^+^ excluder type” preventing the accumulation of sodium ions in leaves. When the above three species were cultivated in farmland with salt damage resulting from the tsunami caused by the Great East Japan Earthquake and farmland where this salt damage had been repaired (control plot), the azuki bean had a lower dry weight in salt-damaged soil than in salt-removed soil, whereas *V. nakashimae* had a similar dry weight in both plots, and *V. riukiuensis* had a higher dry weight in salt-damaged soil than in salt-removed soil (Yoshida *et al.* 2016). Later, Iseki *et al.* (2016) confirmed that *V. riukiuensis* (JP108810) had a higher dry weight when cultivated in a low-salt-treated hydroponic plot (50 mM) than in a salt-free hydroponic plot (Fig. 7A).

Iseki *et al.* (2018) evaluated drought tolerance in 70 accessions from 28 species in the genus *Vigna* and found that *V. minima*, especially JP218938 from Thailand, possessed the highest drought tolerance within section *Angulares* (Fig. 7B). *Vigna minima* is widely found in Southeast Asia (Tomooka *et al.* 2002b), and accessions adapted to various environments, such as sandy drylands, floodplains on the Mekong River, organic wetlands, and dark forests in Cambodia, have been collected (Takahashi *et al.* 2014, 2015b, Tomooka *et al.* 2012, 2013). These accessions possess morphologically diverse leaves ranging from linear to ovate, as well as diverse seeds of different sizes and colors. Since Iseki *et al.* (2018) only used three accessions of *V. minima*, screening a diverse set within the species will likely find accessions with higher drought tolerance than JP218938.

*Vigna hirtella*, *V. minima*, *V. nakashimae*, *V. riukiuensis*, and *V. tenuicaulis* could be used as breeding materials to improve the stress tolerance of the azuki bean with which they can be easily crossed (Fig. 6). Moreover, if genetic loci involved in abiotic stress tolerance or biotic stress resistance are clarified by genetic analysis using segregating populations, then it should be possible to develop stress-tolerant crops more efficiently.

## Conclusion

This review aimed to provide a detailed reference of the genetic resources of the azuki bean. We described the morphology of its wild relatives, the historical transition in their taxonomic treatments, gene pool concept, habitats, and stress tolerance. Understanding taxonomy, evolution, and ecology not only satisfies scientific interests but also makes us aware of any potentially useful traits carried by species. For example, plant breeders have noticed that *V. angularis* var. *nipponensis* and *V. tenuicaulis* possess cold or heat tolerance when they know that both species have been differentiated into different ecological habitats reflected by latitude and altitude (Figs. 4, 5). In addition, it was empirically confirmed that there are accessions with higher heat tolerance in *V. tenuicaulis* than in *V. angularis* var. *nipponensis* in the processes of seed multiplication in summer greenhouses in Tsukuba, Japan, which are periodically subject to extremely high temperatures (near 50°C). As the abiotic stress tolerance of wild plants is a trait acquired through environmental adaptation, we can understand their stress tolerance by observing their habitats. However, it is sometimes difficult to understand biotic stress resistance by observing a species’ habitats because the geographical distribution of accessions with biotic stress resistance is unpredictable (see Fig. 1 in Kondo and Tomooka 2012). We believe that genetic resources conserved in genebanks play an important role in developing our understanding of taxonomy, evolutionary biology, and plant breeding and hope that this review will contribute to these academic fields.

## Author Contribution Statement

YT conceptualized this review. YT and TN wrote the manuscript.

## Figures and Tables

**Table 1.** Classification system of the genus *Vigna*

Subgenus	Section	Common and scientific name of cultigen
***Ceratotropis*** (Piper) Verdc.	***Aconitifoliae*** N. Tomooka et Maxted	**Moth bean:** *Vigna aconitifolia* (Jacq.) Maréchal*
	***Angulares*** N. Tomooka et Maxted	**Azuki bean:** *Vigna angularis* (Willd.) Ohwi et H. Ohashi var. *angularis*
		**Creole bean:** *Vigna reflexo-pilosa* Hayata var. *glabra* (Maréchal, Mascherpa et Stainier) N. Tomooka et Maxted
		**Rice bean:** *Vigna umbellata* (Thunb.) Ohwi et H. Ohashi*
	***Ceratotropis***	**Black gram:** *Vigna mungo* (L.) Hepper var. *mungo*
		**Mungbean:** *Vigna radiata* (L.) Wilczek var. *radiata*
***Haydonia*** (Wilczek) Verdc.	***Glossostylus*** Verdc.	
	***Haydonia***	
	***Microspermae*** Maréchal, Mascherpa et Stainier	
***Lasiospron*** (Benth. emend. Piper) Maréchal, Mascherpa et Stainier.		
***Plectrotropis*** (Schumach.) Bak.	***Plectrotropis***	**Tuber cowpea:** *Vigna vexillata* (L.) A. Rich. var. vexillata* *Vigna vexillata* (L.) A. Rich. var. *macrosperma* Maréchal, Mascherpa et Stainier
	***Pseudoliebrechtsia*** Verdc.	
***Vigna*** Savi	***Catiang*** (DC.) Verdc.	**Cowpea:** *Vigna unguiculata* (L.) Walp. var. *unguiculata*
	***Comosae*** Maréchal, Mascherpa et Stainier	
	***Liebrechtsia*** (De Wild.) Baker fil.	
	***Macrodontae*** Harms	
	***Reticulatae*** Verdc.	
	***Vigna***	**Bambara groundnut:** *Vigna subterranea* (L.) Verdc. var. *subterranea*

*Species in which cultigen and wild are not taxonomically subclassed.

**Table 2.** Described species in the subgenus *Ceratotropis* (Reprint from Takahashi and Tomooka 2020)

Section	Scientific name and original description	Status*
***Aconitifoliae***	***Vigna aconitifolia*** (Jacq.) Maréchal in Bulletin du Jardin Botanique National de Belgique 39:160 (1969)	Validated
	***Vigna aridicola*** N.Tomooka et Maxted in Kew Bulletin 57:613 (2002)	Validated
	***Vigna indica*** T.M. Dixit, K.V. Bhat et S.R. Yadav in Rheedea 21:1 (2012)	Validated
	***Vigna khandalensis*** (Santapau) Sundararagh. et Wadhwa in Current Science 41:429 (1972)	Validated
	***Vigna stipulacea*** (Lam.) Kuntze in Revisio generum plantarum 1:212 (1891)	Validated
	***Vigna subramaniana*** (Babu *ex* Raizada) Raizada in Indian Journal of Forestry 3:133 (1980)	Validated
	***Vigna trilobata*** (L.) Verdc. in Taxon 17:172 (1968)	Validated
***Angulares***	***Vigna angularis*** (Willd.) Ohwi et H. Ohashi in Journal of Japanese Botany 44:29 (1969)	Validated
	***Vigna dalzelliana*** (Kuntze) Verdc. in Kew Bulletin 24:558 (1970)	Validated
	***Vigna exilis*** Tateishi et Maxted in Kew Bulletin 57:625 (2002)	Validated
	***Vigna hirtella*** Ridl. in Journal of the Federated Malay States Museums 10:132 (1920)	Validated
	***Vigna minima*** (Roxb.) Ohwi et H. Ohashi in Journal of Japanese Botany 44:30 (1969)	Validated
	***Vigna nakashimae*** (Ohwi) Ohwi et H. Ohashi in Journal of Japanese Botany 44:30 (1969)	Validated
	***Vigna nepalensis*** Tateishi et Maxted in Kew Bulletin 57:629 (2002)	Validated
	***Vigna reflexo-pilosa*** Hayata in Journal of college of science, Imperial University of Tokyo 30:82 (1911)	Validated
	***Vigna riukiuensis*** (Ohwi) Ohwi et H. Ohashi in Journal of Japanese Botany 44:31 (1969)	Validated
	***Vigna tenuicaulis*** N. Tomooka et Maxted in Kew Bulletin 57:617 (2002)	Validated
	***Vigna trinervia*** (B. Heyne ex Wight et Arn.) Tateishi et Maxted in Kew Bulletin 57:633 (2002)	Validated
	***Vigna umbellata*** (Thunb.) Ohwi et H. Ohashi in Journal of Japanese Botany 44:31 (1969)	Validated
	***Vigna yadavii*** S. P. Gaikwad, Gore, S. D. Randive et Garad in Biodiversity Data Journal 2:e4281 (2014)	Unknown
***Ceratotropis***	***Vigna grandiflora*** (Prain) Tateishi et Maxted in Kew Bulletin 57:632 (2002)	Validated
	***Vigna konkanensis*** Latha, K. V. Bhat, I. S. Bisht, Scariah, K. J. John et Krishnaraj in Journal of Plant Taxonomy and Geography 69:49 (2014)	Unknown
	***Vigna mungo*** (L.) Hepper in Kew Bulletin 11:128 (1956)	Validated
	***Vigna pandeyana*** Gore, S. P. Gaikwad et S. D. Randive in Biodiversity Data Journal 3:e4606 (2015)	Unknown
	***Vigna radiata*** (L.) Wilczek in Flore du Congo Belge et du Ruanda-Urundi 6:386 (1954)	Validated
	***Vigna sahyadriana*** Aitawade, K.V. Bhat et S.R. Yadav in Rheedea 22:1 (2012)	Validated
	***Vigna sathishiana*** Balan et Predeep in Journal of Japanese Botany 92:194 (2017)	Unknown

*Validated: Distinctness as an independent species had been validated using DNA sequences and morphology by the authors of this review.

**Table 3.** Collections of *Vigna angularis* in the genebanks

Institution, Database searched*	Country or region	Number of accessions*
Hokkaido Prefectural Agricultural Experiment Stations, http://www.agri.hro.or.jp/grdb/index.php	Japan	2748
Genetic Resources Research Center, National Agriculture and Food Research Organization (NARO Genebank), https://www.gene.affrc.go.jp/databases-plant_search.php	Japan	2300
Genetic Resources Division, Rural Development Administration, https://genebank.rda.go.kr/eng/uat/uia/actionMain.do	South Korea	1005
World Vegetable Center, https://avrdc.org/our-work/managing-germplasm/	Taiwan	959
Institute of Crop Germplasm Resources, CAAS, https://www.cgris.net/icgr/icgr_english.html	China	839
Australian Grains Genebank, Department of Economic Development Jobs Transport and Resources, https://ausgenebank.agriculture.vic.gov.au/gringlobal/search	Australia	342
Plant Genetic Resources Conservation Unit, Southern Regional Plant Introduction Station, University of Georgia, USDA-ARS, https://www.genesys-pgr.org/	USA	299
N.I. Vavilov Research Institute of Plant Industry, https://www.genesys-pgr.org/	Russia	187
Genebank, Leibniz Institute of Plant Genetics and Crop Plant Research, https://www.genesys-pgr.org/	Germany	84
Embrapa Recursos Genéticos e Biotecnologia, https://www.genesys-pgr.org/	Brazil	29
Embrapa Meio Norte, https://www.genesys-pgr.org/	Brazil	23
Institute of Plant Production n.a. V.Y. Yurjev of UAAS, https://www.genesys-pgr.org/	Ukraine	13
Institute for Agrobotany, https://www.genesys-pgr.org/	Hungary	10
Botanic Garden Meise, https://www.genesys-pgr.org/	Belgium	9
Australian Pastures Genebank, https://apg.pir.sa.gov.au/gringlobal/search	Australia	1

*As per a search conducted in August 2023.

**Table 4.** Active collections of the section *Angulares* in the NARO Genebank, Japan

Scientific name	Number of accessions*	Origin
*Vigna angularis*	2300	Bhutan, China, Japan, Laos, Myanmar, Nepal, South Korea, Taiwan, Vietnam
*Vigna dalzelliana*	7	Myanmar
*Vigna exilis*	41	Thailand
*Vigna hirtella*	106	Laos, Malaysia, Myanmar, Nepal, Sri Lanka, Thailand
*Vigna minima*	243	Cambodia, Indonesia, Laos, Myanmar, Papua New Guinea, Taiwan, Thailand
*Vigna nakashimae*	132	Japan, Korean Peninsula
*Vigna nepalensis*	8	Bhutan, India, Nepal
Vigna reflexo-pilosa**	73	Angola, Cambodia, East Timor, Indonesia, Japan, Laos, Malaysia, Papua New Guinea, Philippines, Vietnam
*Vigna riukiuensis*	154	Japan, Taiwan
*Vigna tenuicaulis*	131	Laos, Myanmar, Thailand
*Vigna trinervia*	85	East Timor, Laos, Malaysia, Myanmar, Sri Lanka
*Vigna umbellata*	536	Angola, Asian countries (omitted), Côte d'Ivoire, Democratic Republic of the Congo, Republic of the Congo, USA

*As per a search conducted in August 2023. **Including syn. *Vigna glabrescens* for its cultigen, *Vigna reflexo-pilosa* var. *glabra*.
